# Dietary proline supplementation alters colonic luminal microbiota and bacterial metabolite composition between days 45 and 70 of pregnancy in Huanjiang mini-pigs

**DOI:** 10.1186/s40104-018-0233-5

**Published:** 2018-01-30

**Authors:** Yujiao Ji, Qiuping Guo, Yulong Yin, Francois Blachier, Xiangfeng Kong

**Affiliations:** 1 National Engineering Laboratory for Pollution Control and Waste Utilization in Livestock and Poultry Production, Key Laboratory of Agro-ecological Processes in Subtropical Region, Institute of Subtropical Agriculture, Chinese Academy of Sciences, Changsha, Hunan 410125 China; 2 Research Center of Mini-pig, Huanjiang Observation and Research Station for Karst Ecosysterms, Huanjiang, Guangxi 547100 China; 3 UMR 914 INRA/AgroParisTech/Universite Paris-Sacaly, Nutrition Physiology and Ingestive Behavior, 75005 Paris, France

**Keywords:** Bacterial metabolites, Colonic microbiota, *L*-proline, Pregnant Huanjiang mini-pigs

## Abstract

**Background:**

Pregnancy is associated with important changes in gut microbiota composition. Dietary factors may affect the diversity, composition, and metabolic activity of the intestinal microbiota. Among amino acids, proline is known to play important roles in protein metabolism and structure, cell differentiation, conceptus growth and development, and gut microbiota re-equilibration in case of dysbiosis.

**Results:**

Dietary supplementation with 1% proline decreased (*P* < 0.05) the amounts of *Klebsiella pneumoniae*, *Peptostreptococcus productus*, *Pseudomonas*, and *Veillonella* spp. in distal colonic contents than that in the control group. The colonic contents of *Butyrivibrio fibrisolvens*, *Bifidobacterium* sp., *Clostridium coccoides*, *Clostridium coccoides-Eubacterium rectale*, *Clostridium leptum* subgroup, *Escherichia coli*, *Faecalibacterium prausnitzii*, *Fusobacterium prausnitzii*, and *Prevotella* increased (*P* < 0.05) on d 70 of pregnancy as compared with those on d 45 of pregnancy. The colonic concentrations of acetate, total straight-chain fatty acid, and total short-chain fatty acids (SCFA) in the proline-supplemented group were lower (*P* < 0.05), and butyrate level (*P* = 0.06) decreased as compared with the control group. Almost all of the SCFA displayed higher (*P* < 0.05) concentrations in proximal colonic contents on d 70 of pregnancy than those on d 45 of pregnancy. The concentrations of 1,7-heptyl diamine (*P* = 0.09) and phenylethylamine (*P* < 0.05) in proximal colonic contents were higher, while those of spermidine (*P* = 0.05) and total bioamine (*P* = 0.06) tended to be lower in the proline-supplemented group than those in the control group. The concentrations of spermidine, spermine, and total bioamine in colonic contents were higher (*P* < 0.05) on d 70 of pregnancy than those measured on d 45 of pregnancy. In contrast, the concentration of phenylethylamine was lower (*P* < 0.05) on d 70 than on d 45 of pregnancy.

**Conclusion:**

These findings indicate that *L*-proline supplementation modifies both the colonic microbiota composition and the luminal concentrations of several bacterial metabolites. Furthermore, our data show that both the microbiota composition and the concentrations of bacterial metabolites are evolving in the course of pregnancy. These results are discussed in terms of possible implication in terms of luminal environment and consequences for gut physiology and health.

## Background

The gut microbiome of pigs is a robust ecosystem inhabited by about 100 trillion bacteria. The importance of the maintenance of host-microbiome symbiosis is underscored by the observation that dysbiotic shifts in microbiota are associated with inflammatory bowel disorders, type 2 diabetes, obesity, and pregnancy metabolic syndrome in humans [1–5]. The gut microbiota metabolizes dietary compounds in both the small and large intestines. The microbiota is present at low concentrations and the transit time is relatively rapid in the small intestine, while the concentration of bacteria is much higher and the transit time is much longer in the large intestine. The dietary compounds that are transferred from the small intestine to the large intestine are undigested or not fully digested compounds, notably undigested carbohydrates and proteins [6]. The metabolic activity of the microbiota allows for the synthesis of various compounds, including short-chain fatty acids (SCFA), indoles, ammonia, gaseous compounds, organic acids, bioamines, and vitamins. Among these various metabolites, some are considered beneficial whereas others are believed to exert deleterious effects on the intestinal mucosa, when present in excess [7]. For instance, among SCFA, butyrate is considered beneficial for the colonic mucosa, because it can exert some anti-inflammatory effects [8]. The SCFA can reach luminal concentrations of 130 mmol/L in the proximal colon [9]. The concentration of SCFA is related to the luminal pH. As weak organic acids, SCFA can decrease the luminal pH and inhibit some pathogenic microorganisms, while increasing the absorption of some nutrients [10]. Bioamines are widely produced by various kinds of bacteria. Although limited amounts of bioamines have no detectable effect on health [11] and participate in the physiology of the host, larger quantities (1.4 g/d) of bioamines can become harmful to humans and livestock [12].

Pregnancy is a biological process involving simultaneous changes in many physiological systems, including microbiome composition [13]. Many nutrients, especially amino acids (AA), are required to sustain a successful pregnancy. For instance, arginine is a conditionally essential AA involved by itself and/or through its metabolites, in spermatogenesis, embryonic survival, fetal growth, as well as maintenance of vascular tone and hemodynamics [14]. Some other amino acid concentrations must be tightly regulated to avoid deleterious effects in some fetal tissues. For instance, limited entry of dietary aspartate and glutamate into blood circulation is required to avoid brain injury in the fetuses [15]. Thus, several evidences indicate that AA play crucial roles in both female and male reproduction [16, 17].

Among AA, it has been shown that proline played several roles in the development of the placenta, conceptus, and fetus [18, 19]. Increasing proline availability in maternal plasma in pigs enhances the concentrations of proline and polyamines in placentae and fetal fluids, and promotes fetal growth [19]. Faure et al. [20] found that dietary proline supply could promote mucin synthesis, re-equilibrate the gut microbiota, and favor mucosal healing in dextran sulfate sodium-treated rats. In addition, proline plays an important role in the metabolism and recycling of nitrogenous compounds in bacteria [10].

Gut microbiota changes markedly from the first to the third trimesters of pregnancy in human beings, with an overall increase in Proteobacteria and Actinobacteria, and reduced richness (i.e., lower species count). Interestingly, the mucosal surfaces of the gut during trimester 3 of pregnancy present low-grade inflammation [21]. Our previous study found that the colonic microbiota displayed spatial and temporal heterogeneity in composition, diversity, and species abundance in different colonic segments from the first to the third trimester of pregnancy [22]. Considering the effect of proline on several physiological functions during pregnancy, and the role of this amino acid as precursors of metabolites with biological effects; the present study was conducted to document the effects of *L*-proline on the colonic luminal microbiota and bacterial metabolite composition (including SCFA and bioamines) in Huanjiang mini-pigs, because pigs can be used as a relevant model for extrapolation to humans [23].

## Methods

### Animals

The present study was carried out in accordance with the Chinese guidelines for animal welfare and experimental protocols and was approved by the Animal Care and Use Committee of Institute of Subtropical Agriculture, Chinese Academy of Sciences [24]. A total of 32 primiparous Huanjiang mini-pigs [average initial body weight (BW) 28.30 ± 0.87 kg] were used in this study.

### Study design

The gilts were obtained from a Huanjiang mini-pig farm located in Huanjiang County, Guangxi province, China. The experimental design consisted of a 2 × 2 factorial arrangement, with two dietary treatments: control (alanine) diet and experimental (proline) diet, and two pregnancy stages: d 45 or 75 of pregnancy. The animals were randomly assigned to one of the two dietary groups on d 15 after mating, with eight pens per group and two sows per pen. The average BW of pigs in the control group and experimental group was 27.84 ± 1.32 kg and 28.82 ± 1.17 kg, respectively. The animals received a same basal diet supplemented with 1% *L*-proline or 0.77% *L*-alanine in the control group. The basal diet was formulated to meet the nutrient requirements and physiological characteristics of Chinese local pigs (Table 1). All animals were housed in 2 m × 3 m pens with cement flooring. Temperature in the room housing the pens was maintained at 22–28 °C. All the pigs had access to drinking water ad libitum from a nipple drinker and were fed twice daily (at 08:30 and 16:30 h) with their diets (approximately 3.0% of BW) from a feeder.

**Table 1:** Composition and nutrient levels of the basal diet (air-dry basis, %)

Ingredients	Content	Nutrient	Levels^b^ , %
Corn	54.00	Digestive energy, MJ/kg	13.40
Soybean meal	12.00	Crude protein	12.04
Rice bran	30.00	Calcium	0.78
Premix^a^	4.00	Phosphorus	0.62
Total	100.00	Arginine	0.65
		Lysine	0.53
		Proline	0.67

^a^One kg of premix contained the following: vitamin A, 10,200 IU; vitamin D_3_, 1600 IU; vitamin E, 75 IU; vitamin K_3_, 75 mg; thiamine, 3 mg; riboflavin, 16 mg; pyridoxine, 3 mg; vitamin B_12_, 0.8 mg; nicotinic acid, 69 mg; *D*-pantothenic acid, 42 mg; folic acid, 4 mg; biotin, 1 mg; chorine, 900 mg; Fe (FeSO_4_·H_2_O), 150 mg; Cu (CuSO_4_·5H_2_O), 11.2 mg; Zn (ZnSO_4_·H_2_O), 63 mg; Mn (MnSO_4_·5H_2_O), 32 mg; I (KI), 1.5 mg; Co (CoCO_3_), 0.3 mg; Se (Na_2_SeO_3_·H_2_O), 0.25 mg; Ca (CaCO_3_), 200 mg; and P (KH_2_PO_4_), 20 mg

^b^Digestive energy was a calculated value, while the others were measured values

### Sample collection

On d 45 or 70 of pregnancy, eight sows per group were weighed and sacrificed using general anesthesia for sample collection at 12 h after the last feeding [25, 26]. After colon recovery, luminal contents of the proximal colon (10 cm at posterior to the ileocecal valve) and the distal colon (10 cm at the end of the colon) were collected and stored at − 80 °C for the extraction of total DNA of microbiota, as well as for determining the concentrations of SCFA and bioamines.

### Colonic microbiota composition analysis

The total DNA was extracted from colonic contents using the QIAamp DNA Stool Mini kit (Qiagen, Hilden, Germany) after chemical and mechanical disruptions [27]. The quality and quantity of DNA were measured using a NanoDrop ND-1000 spectrophotometer (NanoDrop Technologies Inc., Wilmington, DE, USA). Quantitative real-time polymerase chain reaction (qPCR) was performed to determine the number of copies of the 16S rRNA genes of several targeted bacteria [28]. The primers, which were validated previously, are listed in Table 2. The qPCR was performed using SYBR *Premix Ex Taq*™ II kit (TaKaRa Bio Inc., Shiga, Japan) on an ABI 7900HT Fast real-time PCR system (Applied Biosystems, Foster City, CA, USA). The standard curves for all determined bacteria were prepared using plasmid DNA containing each unique 16S rRNA insert. The raw bacterial qPCR data were transformed to lg the number of target genomes per gram of wet digesta.

**Table 2:** Primer pairs for 16S rRNA genes of bacteria

Bacteria	Phylum	Primer sequences (5′ → 3′)	Product size, bp	References
*Bacteroidetes*	Bacteroidetes	F: AGCAGCCGCGGTAAT R: CTAHGCATTTCACCGCTA	184	[62]
*B. fibrisolvens*	Firmicutes	F: CGCATGATGCAGTGTGAAAAGCTC R: CCTCCCGACACCTATTATTCATCG	625	[63]
*Bifidobacterium* sp.	Actinobacteria	F: CTCCTGGAAACGGGTGG R: GGTGTTCTTCCCGATATCTACA	226	[64]
*C. coccoides*	Firmicutes	F: AAATGACGGTACCTGACTAA R: CTTTGAGTTTCATTCTTGCGAA	440	[65]
*C. coccoides*-*E*. *rectale*	Firmicutes	F: CGGTACCTGACTAAGAAGC R: AGTTTYATTCTTGCGAACG	429	[65]
*C. leptum* subgroup	Firmicutes	F: GCACAAGCAGTGGAGT R: CTTCCTCCGTTTTGTCAA	239	[66]
*E. coli*	Proteobacteria	F: GACCTCGGTTTAGTTCACAGA R: CACACGCTGACGCTGACCA	96	[67]
*F. prausnitzii* ^a^	Firmicutes	F: AATTCCGCCTACCTCTGCACT R: GGAGGAAGAAGGTCTTCGG	248	[68]
Firmicutes	Firmicutes	F: GTCAGCTCGTGTCGTGA R: CCATTGTAKYACGTGTGT	179	[69]
*F*. *prausnitzii*^b^	Bacteroidetes	F: CCCTTCAGTGCCGCAGT R: GTCGCAGGATGTCAAGAC	158	[70]
*K. pneumoniae*	Proteobacteria	F: CCTGGATCTGACCCTGCAGTA R: CCGTCGCCGTTCTGTTTC	165	[71]
*Lactobacillus* sp.	Firmicutes	F: TACATCCCAACTCCAGAACG R: AAGCAACAGTACCACGACC	116	[72]
*M. elsdenii*	Firmicutes	F: GACCGAAACTGCGATGCTAGA R: TCCAGAAAGCCGCTTTCGCCACT	128	[73]
*P. aeruginosa*	Proteobacteria	F: TCCAAGTTTAAGGTGGTAGGCTG R: CTTTTCTTGGAAGCATGGCATC	117	[74]
*P. productus*	Firmicutes	F: AACTCCGGTGGTATCAGATG R: GGGGCTTCTGAGTCAGGTA	268	[67]
*Pseudomonas*	Proteobacteria	F: GAGTTTGATCCTGGCTCAG R: CCTTCCTCCCAACTT	440	[75]
*Prevotella*	Bacteroidetes	F: CACRGTAAACGATGGATGCC R: GGTCGGGTTGCAGACC	513	[64]
*Roseburia*	Firmicutes	F: TACTGCATTGGAAACTGTCG R: CGGCACCGAAGAGCAAT	230	[76]
*S. ruminantium*	Firmicutes	F: TGCTAATACCGAATGTTG R: TCCTGCACTCAAGAAAGA	513	[77]
*Veillonella* spp.	Firmicutes	F: A(C/T)CAACCTGCCCTTCAGA R: CGTCCCGATTAACAGAGCTT	335	[70]

^a^ *Faecalibacterium prausnitzii*

^b^ *Fusobacterium prausnitzii*

### Bacterial metabolite analysis

The SCFA, including straight-chain fatty acids (acetate, propionate, butyrate, and pentanoate) and branched-chain fatty acids (BCFA; isobutyrate, and isopentanoate) were analyzed by gas chromatography as described by Zhou et al. [29]. The bioamines, including 1,7-heptyl diamine, cadaverine, phenylethylamine, putrescine, tryptamine, tyramine, spermidine, and spermine were analyzed by high-performance liquid chromatography as described by Xu et al. [30].

### Statistical analysis

The data were analyzed by a mixed-effects model using SAS version 8.2 (SAS Institute Inc., Cary, NC, USA). Diet, pregnancy stage, and their interaction were included in the statistical model. Effects were considered statistically significant at *P* < 0.05, while a tendency was considered for 0.05 ≤ *P* < 0.10.

## Results

### Microbiota composition

Tables 3 and 4 summarized the effects of diets and pregnancy stages on microbiota composition of proximal and distal colonic contents, respectively, from pregnant Huanjiang mini-pigs. In proximal colonic contents, the proportion of *Prevotella* was lower (*P* < 0.05), but Firmicutes/Bacteroidetes (F/B) ratio was higher (*P* < 0.05) in the proline-supplemented group than in the control group. The proportions of *Butyrivibrio fibrisolvens*, *Bifidobacterium* sp., *Clostridium coccoides*, *Escherichia coli*, *Faecalibacterium prausnitzii*, *Fusobacterium prausnitzii*, and *Prevotella* were higher (*P* < 0.05) on d 70 of pregnancy than on d 45 of pregnancy. The proportion of *Clostridium leptum* subgroup (*P* = 0.09), Firmicutes (*P* = 0.07), *Peptostreptococcus productus* (*P* = 0.08), and F/B ratio (*P* = 0.07) displayed an increasing trend with pregnancy progress. The proportion of *C. coccoides-Eubacterium rectale* and Firmicutes, and F/B ratio were changed (*P* < 0.05) owing to diet × stage interactions, and a trend was measured for *Pseudomonas* (*P =* 0.06).

**Table 3:** Bacteria groups or species in proximal colonic contents of pregnant Huanjiang mini-pigs (lg bacteria cells/g wet weight)

Bacteria	Control group		Proline group		SEM	*P-*values		
	45 d	70 d	45 d	70 d		Diet	Day	Diet × Day
Bacteroidetes	11.18	11.21	10.96	11.06	0.23	0.18	0.63	0.78
*B. fibrisolvens*	8.97	9.54	8.73	9.33	0.26	0.20	0.003	0.93
*Bifidobacterium* sp.	8.97	9.98	8.73	9.98	0.24	0.71	0.001	0.70
*C*. *coccoides*	10.23	10.77	10.01	10.76	0.25	0.47	0.001	0.54
*C*. *coccoides*-*E. rectale*	9.94	10.31	9.68	10.09	0.24	0.11	0.90	0.01
*C*. *leptum* subgroup	10.56	10.67	10.29	10.66	0.23	0.30	0.09	0.33
*E. coli*	9.93	10.31	9.77	10.30	0.22	0.53	0.003	0.56
*F. prausnitzii* ^1^	10.50	10.75	10.28	10.77	0.22	0.41	0.01	0.34
Firmicutes	11.10	11.04	10.83	11.33	0.21	0.94	0.07	0.03
*F. prausnitzii* ^2^	8.55	10.75	8.15	10.77	0.29	0.41	0.01	0.34
*K. pneumoniae*	7.57	7.53	7.72	7.51	0.24	0.67	0.39	0.56
*Lactobacillus* sp*.*	10.27	10.53	10.25	10.61	0.31	0.90	0.22	0.84
*M. elsdenii*	8.23	7.55	7.72	7.88	0.33	0.75	0.36	0.14
*P. aeruginosa*	8.73	8.43	8.93	8.72	0.25	0.15	0.13	0.76
*P. productus*	7.87	8.07	7.81	8.04	0.22	0.68	0.08	0.87
*Pseudomonas*	6.80	7.24	7.03	6.99	0.22	0.92	0.12	0.06
*Prevotella*	9.01	9.86	8.59	9.23	0.30	0.03	0.004	0.67
*Roseburia*	8.31	8.47	7.95	8.37	0.28	0.27	0.16	0.54
*S. ruminantium*	7.26	7.53	7.03	7.17	0.28	0.16	0.34	0.74
*Veillonella* spp.	7.21	6.83	6.88	6.90	0.27	0.48	0.34	0.29
F/B ratio	0.99	0.99	0.99	1.02	0.05	0.02	0.07	0.01

F/B ratio Firmicutes/Bacteroidetes ratio

**Table 4:** Bacteria groups or species in distal colonic contents of pregnant Huanjiang mini-pigs (lg bacteria cells/g wet weight)

Item	Control group		Proline group		SEM	*P-*values		
	45 d	70 d	45 d	70 d		Diet	Day	Diet × Day
*Bacteroidetes*	11.04	11.03	10.91	11.11	0.23	0.84	0.47	0.46
*B. fibrisolvens*	8.72	9.12	8.63	9.22	0.23	0.98	0.001	0.49
*Bifidobacterium* sp.	9.17	9.99	9.39	9.53	0.27	0.52	0.02	0.08
*C*. *coccoides*	10.28	10.72	10.19	10.61	0.21	0.40	0.001	0.94
*C*. *coccoides*-*E. rectale*	9.79	10.12	9.81	10.08	0.23	0.95	0.04	0.84
*C*. *leptum* subgroup	9.98	10.42	9.97	10.19	0.24	0.42	0.04	0.44
*E. coli*	9.89	10.14	9.82	10.21	0.22	0.98	0.02	0.59
*F. prausnitzii* ^1^	10.10	10.33	10.11	10.35	0.20	0.88	0.03	0.94
Firmicutes	10.77	11.08	10.70	10.95	0.21	0.38	0.02	0.78
*F. prausnitzii* ^2^	7.92	8.43	7.91	8.29	0.23	0.57	0.003	0.65
*K. pneumoniae*	7.66	7.39	7.15	7.43	0.22	0.06	0.98	0.03
*Lactobacillus* sp*.*	9.82	10.10	9.73	9.97	0.31	0.66	0.29	0.93
*M. elsdenii*	8.12	7.77	7.84	7.84	0.31	0.68	0.48	0.49
*P. aeruginosa*	8.70	8.68	9.13	8.52	0.24	0.37	0.04	0.06
*P. productus*	7.70	8.23	7.65	7.77	0.20	0.02	0.01	0.06
*Pseudomonas*	7.23	6.81	6.60	6.82	0.26	0.08	0.56	0.07
*Prevotella*	8.61	9.25	8.18	8.90	0.31	0.12	0.01	0.87
*Roseburia*	7.98	8.07	8.06	8.12	0.28	0.75	0.69	0.93
*S. ruminantium*	7.03	7.12	7.01	7.37	0.29	0.61	0.31	0.54
*Veillonella* spp.	7.19	6.98	6.83	6.78	0.25	0.09	0.43	0.63
F/B ratio	0.98	1.00	0.98	0.99	0.06	0.46	0.09	0.21

F/B ratio Firmicutes/Bacteroidetes ratio

In distal colonic contents, the proportion of *Klebsiella pneumoniae (P =* 0.06)*, P. productus* (*P <* 0.05)*, Pseudomonas* (*P =* 0.08)*,* and *Veillonella* spp. (*P =* 0.09) tended to be lower in the proline-supplemented group than in the control group. The proportion of most bacteria, including *B. fibrisolvens, Bifidobacterium* sp., *C. coccoides*, *C. coccoides-E. rectale, C. leptum* subgroup, *E. coli, Faecalibacterium prausnitzii*, Firmicutes, *Fusobacterium prausnitzii*, *P. productus,* and *Prevotella* increased (*P* < 0.05), whereas that of *Pseudomonas aeruginosa* decreased (*P* < 0.05) with the progress of pregnancy. The F/B ratio displayed an increasing trend (*P* = 0.09) with the progress of pregnancy. The proportion of *Bifidobacterium* sp. (*P* = 0.09), *K. pneumoniae* (*P* < 0.09), *P. aeruginosa* (*P* = 0.06), *P. productus* (*P* = 0.06), and *Pseudomonas* (*P* = 0.07) changed owing to the diet × stage interactions.

### The SCFA concentrations

The SCFA concentrations in colonic contents of pregnant Huanjiang mini-pigs are summarized in Table 5. In proximal colonic contents, the concentrations of acetate, total straight-chain fatty acids, and total SCFA were lower (*P* < 0.05) in the proline-supplemented group. The concentrations of butyrate tended to be decreased (*P* = 0.06) in the proline-supplemented group, when compared with the control group. Almost all of the SCFA in proximal colonic contents presented higher (*P* < 0.05) concentrations on d 70 of pregnancy than on d 45 of pregnancy. The concentrations of acetate (*P* = 0.099), butyrate (*P* = 0.05), isovalerate (*P* = 0.09), total straight-chain fatty acids (*P* = 0.06), and total SCFA (*P* = 0.05) were changed, or tended to be changed, owing to the diet × stage interactions. In distal colonic contents, the concentrations of isobutyrate and total BCFA were higher (*P* < 0.05) on d 70 of pregnancy than on d 45 of pregnancy. Proline supplementation, however, did not affect the concentrations of SCFA.

**Table 5:** Short-chain fatty acid concentrations in colonic contents of pregnant Huanjiang mini-pigs (mg/g)

Item	Control group		Proline group		SEM	*P-*values		
	45 d	70 d	45 d	70 d		Diet	Day	Diet × Day
Proximal colonic contents								
Acetate	16.72	34.24	14.70	22.76	1.07	0.02	< 0.01	0.099
Propionate	5.95	12.56	6.36	9.44	0.71	0.28	< 0.01	0.16
Isobutyrate	0.23	0.92	0.28	0.71	0.19	0.41	< 0.01	0.17
Butyrate	3.64	6.69	3.68	4.18	0.51	0.06	0.010	0.05
Isovalerate	0.19	0.70	0.21	0.50	0.16	0.15	< 0.01	0.09
Valerate	0.43	1.43	0.44	1.16	0.24	0.35	< 0.01	0.34
Total BCFA	0.42	1.61	0.49	1.21	0.25	0.28	< 0.01	0.13
Total straight-chain fatty acids	26.75	54.92	25.18	37.54	1.27	0.02	< 0.01	0.06
Total SCFA	27.17	56.53	25.67	38.74	1.29	0.03	< 0.01	0.05
Distal colonic contents								
Acetate	10.48	9.07	10.38	10.02	0.60	0.60	0.28	0.52
Propionate	4.01	3.81	4.00	4.60	0.38	0.24	0.54	0.24
Isobutyrate	0.36	0.49	0.34	0.48	0.13	0.78	0.01	0.92
Butyrate	2.94	2.95	2.93	2.71	0.38	0.71	0.75	0.73
Isovalerate	0.33	0.39	0.31	0.34	0.13	0.34	0.25	0.62
Valerate	0.48	0.50	0.41	0.51	0.14	0.57	0.17	0.39
Total BCFA	0.69	0.88	0.66	0.82	0.18	0.53	0.03	0.84
Total straight-chain fatty acids	17.90	16.33	17.72	17.85	0.76	0.61	0.59	0.52
Total SCFA	18.59	17.21	18.38	18.67	0.77	0.64	0.69	0.54

### Bioamine contents

The bioamine concentrations in colonic contents of pregnant Huanjiang mini-pigs are summarized in Table 6. In proximal colonic contents, the concentrations of 1,7-heptyl diamine (*P* = 0.09) and phenylethylamine (*P* < 0.05) tended to and were significantly higher in the proline-supplemented group, respectively, whereas those of spermidine (*P* = 0.05) and total bioamine (*P* = 0.06) were lower or tended to be lower than those in the control group. The concentrations of spermidine, spermine, and total bioamine were higher (*P* < 0.05) on d 70 of pregnancy, whereas that of phenylethylamine (*P* < 0.05) was lower than those on d 45 of pregnancy. The concentrations of 1,7-heptyl diamine, phenylethylamine, spermine, and tryptamine displayed differences (*P* < 0.05) according to diet × stage interactions, as well as total bioamine concentration (*P* = 0.096). In distal colonic contents, the concentrations of 1,7-heptyl diamine (*P* = 0.07) and tryptamine (*P* < 0.05) were higher in the proline-supplemented group, whereas those of cadaverine (*P* < 0.05), phenylethylamine (*P* < 0.05), and tyramine (*P* = 0.098) were lower than those in the control group. The concentrations of spermidine (*P* < 0.05), spermine (*P* < 0.05), tryptamine (*P* < 0.05), and total bioamine (*P* = 0.08) were higher, but cadaverine and phenylethylamine were lower (*P* < 0.05) on d 70 than on d 45 of pregnancy. The concentrations of cadaverine, phenylethylamine, putrescine, and tryptamine displayed differences (*P* < 0.05) according to diet × stage interactions.

**Table 6:** Bioamine concentrations in colonic contents of pregnant Huanjiang mini-pigs (μg/g)

Items	Control group		Proline group		SEM	*P-* values		
	45 d	70 d	45 d	70 d		Diet	Day	Diet × Day
Proximal colonic contents								
1,7-heptyl diamine	2.22	2.36	2.95	2.28	0.26	0.09	0.15	0.04
Cadaverine	13.61	12.77	12.99	16.67	0.98	0.50	0.56	0.36
Phenylethylamine	6.67	8.64	12.99	6.36	0.44	0.001	< 0.01	< 0.01
Putrescine	20.30	24.38	21.83	19.12	0.99	0.45	0.78	0.18
Spermidine	35.97	67.63	30.96	44.35	1.64	0.05	0.005	0.19
Spermine	5.71	15.79	9.15	10.67	0.83	0.64	0.006	0.03
Tryptamine	1.46	2.14	2.46	1.03	0.39	0.89	0.35	0.02
Tyramine	2.82	2.53	2.02	1.79	0.45	0.15	0.61	0.96
Total bioamine	101.90	151.18	98.32	106.42	2.15	0.06	0.03	0.096
Distal colonic contents								
1,7-heptyl diamine	1.63	1.24	1.80	2.06	0.32	0.07	0.78	0.22
Cadaverine	15.81	6.23	5.71	6.81	0.80	0.01	0.02	0.005
Phenylethylamine	10.09	4.47	5.70	4.57	0.54	0.011	< 0.01	0.008
Putrescine	13.87	10.51	8.84	14.30	0.65	0.56	0.33	0.001
Spermidine	29.54	33.79	22.67	38.67	1.21	0.79	0.02	0.13
Spermine	7.31	9.92	7.86	9.95	0.66	0.79	0.046	0.81
Tryptamine	0.28	0.23	0.17	1.31	0.23	0.002	0.001	< 0.01
Tyramine	1.71	1.87	0.96	1.67	0.33	0.098	0.12	0.32
Total bioamine	64.82	73.97	68.31	79.33	1.47	0.42	0.08	0.86

## Discussion

Several studies revealed that the indigenous microbiota in the gut play important roles in the metabolism and recycling of nitrogenous compounds, including AA [10, 20]. Furthermore, changes in gut microbiota of pregnant human and animals have been documented in recent years [13, 23, 31]. The present study indicates that dietary supplementation with proline affects the colonic luminal microbiota and bacterial metabolite composition in Huanjiang mini-pigs. In addition, our study confirms that the composition of bacteria in the colon, as well as the luminal environment, differ according to the stage of pregnancy.

Pregnancy is a time of dramatic host remodeling for the mother, and may be partly viewed as the development of adaptive processes in a context of major new physiological constraints. Our previous study showed that during pregnancy, both the body weight and fat over lean mass ratio increased in the Huanjiang mini-pigs [32]. In agreement with the above, it has been reported that pregnant female Ossabaw mini-pigs displayed higher body weight, notably due to fat deposition [33], which was association with higher levels of triglyceride and very low-density lipoprotein [34], and lower levels of high-density lipoprotein cholesterol, and low-density lipoprotein cholesterol [35].

A previous study showed that the pregnancy stage and diet composition may affect gut microbial composition [36]. The present study confirms these results and elucidates that the proportion of *Prevotella* increased with progress of pregnancy. This is consistent with the study of Collado and Isolauri [3], who reported that the abundances of *Bacteroides-Prevotella* group, *Clostridium*, and *Staphylococcus* increased from first trimester to third trimester of normal-weight and overweight pregnant women. Moreover, Santacruz et al. [37] confirmed that the proportion of Firmicutes, especially *Clostridium* clusters, is associated with excessive BW and obesity in human subjects. Similar to the obese human subjects, the sows at d 70 of pregnancy had increased proportions of *C. coccoides*, *C. leptum* subgroup, *E. coli*, *Faecalibacterium prausnitzii*, *P. productus*, and Firmicutes, as compared with those at d 45 of pregnancy. The increased proportion of Firmicutes is considered to affect the metabolic potential of the gut microbiota and enhance the capacity of the body to harvest energy from the diet [38]. In addition, colonization of germ-free mice with the butyrate-producing bacteria *B. fibrisolvens* rescued colonic epithelia from the energy starvation status [39]. Based on our results, it appeared that some bacteria involved in indigestible carbohydrate fermentation increased by d 70 of pregnancy, thereby allowing the production of more SCFA and increased energy recycling for the pregnant sows and their fetuses.

Bacterial cross-feeding has a huge impact on the final balance of the SCFA production, absorption, and efficient exploitation of the substrates in the gut [40]. The vast majority of acetate in the body is produced by the gut microbiota, and the total fecal propionate concentration is linked to the relative abundance of Bacteroidetes and Firmicutes [41]. Butyrate produced by the gut microbiota is dependent on butyrate-producers, such as *Faecalibacterium*, *Eubacterium*, and *Roseburia* [42]. In accordance with some data on the evolution of the microbiota composition during pregnancy, SCFA presented higher concentrations in proximal colonic contents. In the distal colonic contents recovered at d 70 of pregnancy. Isobutyrate and total BCFA concentrations in the distal colonic contents were higher at d 70 than at d 45 of pregnancy. As BCFA concentrations are considered as indicators of protein catabolism by the microbiota in the luminal intestinal content [43], these results are suggestive of increased protein fermentation in the distal colon at d 70 of pregnancy as compared with that at d 45. The present study shows that the proportion of saccharolytic bacteria generating SCFA [44], including *C. coccoides*, *C. coccoides-E. rectale*, and *C. leptum* subgroups, increases similarly when compared with the Firmicutes phylum. *Faecalibacterium prausnitzii*, *Fusobacterium*, and *Clostridium* are known butyrate producers [45–48]. *Prevotella* had been proposed to enhance calorie extraction from resistant starches, oligosaccharides, and other indigested carbohydrates [49], and its concentration is associated with increased colonic SCFA. In addition to participating in the digestive process, microbiota allows local synthesis of SCFA, which are used as energy substrates by the host [50]. Parts of SCFA are absorbed and metabolized by the colonocytes, while the unmetabolized portion can enter diverse carbohydrates and lipid metabolic routes in the peripheral tissues. While most of the butyrate produced by the microbiota is metabolized in enterocytes/colonocytes during its transfer from the intestinal lumen to the bloodstream, propionate mainly incorporates into the gluconeogenesis pathway, while acetate is mostly metabolized into the lipid biosynthesis pathway [41]. Collectively, the increased SCFA production and absorption may provide additional nutrients for pregnant sows; however, additional work outside of the scope of the present study is necessary to test this hypothesis.

The AA serve not only as protein building bricks but also act as energy substrates, signaling molecules, and/or as precursors for bioactive compounds [51]. Thus, AA intervene in the regulation of diverse physiological process related to the reproductive functions, ranging from spermatogenesis to oocyte fertilization and embryo implantation [52]. In the large intestine, AA are not absorbed to any significant extent by the colonic mucosa (except in the neonatal period), and thus are mostly metabolized by the microbiota into various intermediary and end products [53]. The present study showed that dietary proline supplementation decreased the proportion of *Prevotella* in the proximal colonic contents, and the proportions of *K. pneumoniae* and *P. productus* in the distal colonic contents. These bacterial species can metabolize carbohydrates, especially indigestible fiber [54]. *Klebsiella pneumoniae*, which is the most significant member of Enterobacteriaceae and *Peptostreptococcus productus*, are predominant for the utilization of glutamate or tryptophan [52]. Ren et al. [55] reported that dietary proline supplementation confers a positive immune effect in porcine circovirus-infected pregnant and non-pregnant mice.

Proteins are degraded by the bacterial protease and peptidase activities, and the AA released from the proteins can be precursors of various bioamines in the colon via specific AA decarboxylation pathways by specific bacteria [56]. In bacteria, the bioamines are involved in many processes related to transcription, translation, growth, metabolism, and other functions, including improved acid resistance, protection from oxidative stress and host immunological defenses [56–60]. For instance, *E. coli* synthesized cadaverine during anaerobic growth at low pH in the presence of its precursor lysine [61]. Several bioamine-producing taxa belong to *Prevotella* and *Megasphaera* [60]. This may explain why the concentrations of spermidine, spermine, and total bioamines in colonic contents increased with the progress of pregnancy.

The way by which supplemental proline exerts its effect on colonic microbiota remains elusive, considering that the capacity of the small intestine for the absorption of amino acids is high. In other words, the dietary proline originating from the dietary proteins and supplement is most likely large, if not totally absorbed by the small intestinal epithelium. Thus, we propose that the effects of dietary proline on the colonic ecosystem would be dependent, at least in part, on the effect of this AA on the microbiota composition and metabolic activity in the small intestine; such effects presumably affect the large intestinal luminal environment. This hypothesis is worthy of testing in future experiments.

## Conclusion

In conclusion, the present study indicates that *L*-proline supplementation modifies the colonic microbiota composition and the luminal concentrations of several bacterial metabolites. Furthermore, our data show that both the microbiota composition and the concentrations of bacterial metabolites are evolving in the course of pregnancy. The changes in the luminal environment associated with these changes in microbiota composition need to be evaluated in terms of beneficial over deleterious effects for the colonic mucosa and for peripheral tissues in the mother and the fetuses.

## Abbreviations

AA: Amino acids
BCFA: Branched-chain fatty acids
qPCR: Quantitative real-time polymerase chain reaction
SCFA: Short-chain fatty acids
